# Intraclass Image Augmentation for Defect Detection Using Generative Adversarial Neural Networks

**DOI:** 10.3390/s23041861

**Published:** 2023-02-07

**Authors:** Vignesh Sampath, Iñaki Maurtua, Juan José Aguilar Martín, Ander Iriondo, Iker Lluvia, Gotzone Aizpurua

**Affiliations:** 1 Smart and Autonomous System Unit, Tekniker, Member of Basque Research & Technology Alliance, 20600 Eibar, Spain; 2 Department of Design and Manufacturing Engineering, School of Engineering and Architecture, University of Zaragoza, 50009 Zaragoza, Spain

**Keywords:** class imbalance, convolutional neural network, defect detection, GAN, image augmentation, limited data, synthetic images, transfer learning

## Abstract

Surface defect identification based on computer vision algorithms often leads to inadequate generalization ability due to large intraclass variation. Diversity in lighting conditions, noise components, defect size, shape, and position make the problem challenging. To solve the problem, this paper develops a pixel-level image augmentation method that is based on image-to-image translation with generative adversarial neural networks (GANs) conditioned on fine-grained labels. The GAN model proposed in this work, referred to as Magna-Defect-GAN, is capable of taking control of the image generation process and producing image samples that are highly realistic in terms of variations. Firstly, the surface defect dataset based on the magnetic particle inspection (MPI) method is acquired in a controlled environment. Then, the Magna-Defect-GAN model is trained, and new synthetic image samples with large intraclass variations are generated. These synthetic image samples artificially inflate the training dataset size in terms of intraclass diversity. Finally, the enlarged dataset is used to train a defect identification model. Experimental results demonstrate that the Magna-Defect-GAN model can generate realistic and high-resolution surface defect images up to the resolution of 512 × 512 in a controlled manner. We also show that this augmentation method can boost accuracy and be easily adapted to any other surface defect identification models.

## 1. Introduction

Nondestructive testing (NDT) plays an essential role in industrial applications that can benefit directly from computer vision algorithms. They are widely employed in the manufacturing sector to detect defects, including scratches, flaws, pores, leaks, fractures, and cracks. In addition to impairing the aesthetic of the corresponding object, these defects on the object surface may also have a negative impact on quality control or even pose serious manufacturing safety risks [1]. The traditional procedures of performing NDT methods are more susceptible to the effects of human factors, which can result in different outcomes for the same test. Therefore, the incorporation of automation and computer vision techniques is desirable. Computer vision models excel at inspecting object details and defect detection tasks because of their speed, accuracy, and repeatability.

MPI is used to inspect a wide variety of manufactured products in different forms including castings, forgings, and weldments. The principle of magnetism is used in MPI to find defects in magnetic materials such as steel, iron, nickel, cobalt, etc. The first step in MPI is to magnetize the component parallel to its surface that is to be inspected. In the case of defects on or near the surface of the component, the defects create a leakage field. Then, the iron particles in wet suspended form are applied onto the component. In the places of leakage fields, the particles are attracted and clustered. The defects can provide visible indications under ultraviolet light [2]. There are several factors that influence the effectiveness of MPI. The main factors include:

1. Part geometry: The shape and size of the part being inspected can affect the effectiveness of the inspection. For example, it may be more difficult to detect defects in thin or small parts compared to larger or thicker parts.
2. Material properties: The material properties of the part being inspected can also affect the effectiveness of the inspection. For example, nonferromagnetic materials may not be suitable for magnetic particle inspection.
3. Surface finish: a rough or uneven surface can make it more difficult to detect defects using magnetic particle inspection.
4. Magnetizing force: The strength of the magnetizing force applied during the inspection can affect the sensitivity of the inspection. A stronger magnetizing force may be more effective at detecting smaller defects.
5. Particle size and type: The size and type of magnetic particles used in the inspection can also affect the effectiveness of the inspection. Smaller particles may be more sensitive to defects but may be more difficult to see.
6. Light intensity: the intensity of the light used to illuminate the magnetic particles can affect the visibility of the particles and the ability to detect defects.

Collecting defective images with different combinations of these factors (intraclass variations) at a large scale is expensive due to the low possibility of defecting occurrence [3]. It leads to several difficulties in acquiring defect data with a high range of variability and hence poor generalization ability of a defect detection model. One of the most challenging tasks in developing a defect detection model is to improve its generalization ability.

**Figure 1:**
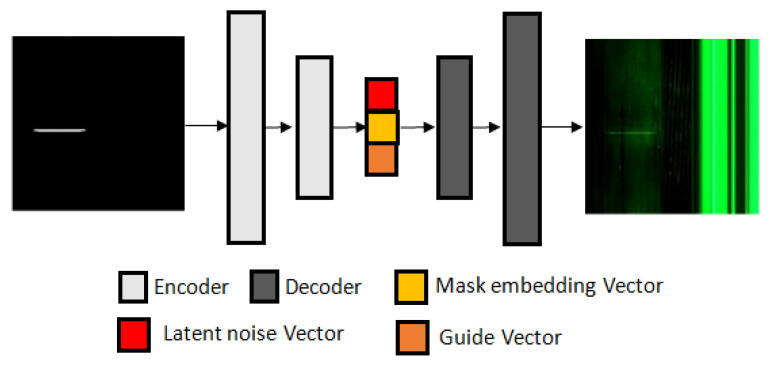
Structure of the proposed Magna-Defect-GAN.

To address the issue of the insufficient generalization ability of a defect detection algorithm caused by the limited data problem, in this paper, an improved conditional mask-to-image translation GAN-based data augmentation method is proposed. GAN-based intraclass augmentation is used to artificially increase the size and diversity of the dataset, which can improve the performance of the model. Intraclass image augmentation refers to the process of applying various types of data augmentation techniques to images within the same class in order to increase the variability of the training dataset. This can help to improve the generalization performance of a machine learning model by providing it with more examples of the same class with different variations. Unlike previous work, for our generator, we use a U-Net-based network, we couple the mask embedding vector with the latent noise vector and the discrete fine-grained guide labels (Figure 1), and for our discriminator, we use a PatchGAN classifier [4]. Coupling embedding vectors with fine-grained guide labels and latent noise vectors leads to conditioning the data generation process in a controlled manner. With the mask, our Magna-Defect-GAN model can generate diverse defect images, such as by changing defect size, shape, location, position, etc. We also allow more diversity, such as by changing the background, thickness, and brightness of the defects.

To verify the effectiveness of the proposed network, we acquired a defect dataset using a line scan camera from an MPI apparatus, located at Erreka Fastening solutions, in a controlled manner. The Magna-Defect-GAN model is trained for augmenting data samples. The defect detection accuracies of a convolutional neural network (CNN) model before and after data augmentation are compared. The experimental results show that the Magna-Defect-GAN is more robust in generating controllable realistic and high-resolution defect images than other existing GAN models.

The key contributions of this paper are as follows: (1) we present a surface defect dataset acquired from a line scan camera that is essential for defect detection in cylindrical objects; (2) we propose combinations of the mask, latent vector, and guide vectors (background, thickness, and brightness vectors) as a means of controlling the conditions of the synthesized images; (3) we present a novel conditional mask-to-image GAN that utilizes the interpretable guide vectors, and the Magna-Defect-GAN is employed to augment training data at pixel level; and (4) we validate the effectiveness of the proposed pixel-level data augmentation by training the CNN model with various training schemes using synthetic and original data. The defect detection model trained by the combination of original and augmented data alleviates the problem of overfitting and overcomes all biases present in a limited dataset. Several forms of biases in the limited dataset such as background, lighting, defect position, shape, size, etc., are drastically lessened with the help of GAN-generated synthetic images.

The remainder of the paper is structured as follows: In the next section, existing work on the classical and GAN-based data augmentation methods are described in detail. In Section 3, an experimental platform for defect image acquisition is established in the laboratory. The GAN-based data augmentation models are built to generate synthetic images for enhancing intraclass diversity in a limited data regime, and some comparative experiments are performed in Section 4 to test the efficacy of the GAN-based data augmentation. The effectiveness of the Magna-Defect-GAN-based data augmentation is examined in Section 5, and the findings are reported. In Section VI, conclusions are drawn.

## 2. Related Work

There are thousands of parameters in even a lightweight CNN model that need to be trained. When employing deep CNN models with numerous layers or when working with a small number of training images, there is a risk of overfitting. The most widely used method to reduce overfitting is data augmentation, which artificially inflates the dataset size. By exposing the defect detection model to a wider range of variations in the data, data augmentation can help the model learn to generalize better and reduce overfitting. For example, if the model is trained on images of defects that all have the same orientation, it may not be able to recognize defects that have a different direction. However, if the model is also trained on images of defects that are rotated or flipped, it may be able to recognize defects in a wider range of orientations. This encompasses classic augmentation techniques such as affine and color transformations [5]. Even though classic augmentation techniques serve as an implicit regularization, they are limited in augmentation diversity. Several methods have been introduced to increase the effectiveness of data augmentation. Zhong et al. [6] proposed a random erasing augmentation technique to make sure that the CNN pays attention to the entire image rather than its subset. Random erasing works by discarding a random n × m rectangle patch in an image and masking it with random values. The disadvantage of using random erasing in defect identification applications is that it is not always a label-preserving augmentation. Moreno-Barea et al. [7] injected random noise into images that can help the model to learn more robust features. Combining different augmentation techniques can result in an enormously expanded dataset size. However, it is not ensured to be beneficial. Cubuk et al. [8] proposed an autoaugment policy based on the reinforcement learning algorithm to search for an optimal combination of augmentation techniques. Perez and Wang [9] developed an augmentation method based on the neural style transfer algorithm, which employs neural nets to transfer style and classify the image.

Recently, GAN-based data augmentation gained momentum in the field of computer vision [10]. GAN models can be used to create synthetic images such that they retain similar characteristics to the original training data. One way to use GAN-generated synthetic images in defect detection is to train a defect detection model on a combination of real and synthetic images. Using GANs to generate synthetic images can be useful for augmenting the training dataset for a defect detection model. By adding synthetic images to the training dataset, it is possible to increase the diversity of the dataset and improve the generalization ability of the model. The objective of GAN-based augmentation is to generate synthetic images to increase diversity and the amount of the original dataset. Several modifications of the original GAN [11] have been proposed to improve the performance and stability of GAN training including DCGAN [12], Pro-GAN [13], LAPGAN [14], GRAN [15], D2GAN [16], SinGAN [17], and MADGAN [18]. However, these GAN models have a limitation in that the generated synthetic images cannot be controlled. Conditional variants of GANs such as cGAN [4], ACGAN [19], VACGAN [20], info-GAN [21], and SCGAN [22] have been proposed to overcome the limitation. GAN models have proved to excel at several other computer vision tasks including image super-resolution [23], image denoising [24], and text to image synthesis [25]. In the area of manufacturing, image-to-image translation is the most pertinent use of GAN.

In 2016, P. Isola et al. [26] developed a conditional variant of GAN called Pix2Pix (pixel to pixel) GAN as a general solution to image-to-image translation tasks. In this case, the generator takes an image from one domain and is tasked to convert it into an image in another domain by minimizing reconstruction as well as the adversarial loss. Several variants of Pix2Pix GAN have been proposed to enhance the quality of the translated images. To reduce the blurriness of the translated images, Wang et al. [27] replaced the reconstruction loss with a feature-matching loss. Unsupervised variants of image-to-image translation GANs such as Disco-GANs [28] and Cycle-GANs [29] were proposed.

The ability to generate industrial images with defects in a controlled manner is highly desirable by the industry 4.0 machine learning community. In particular, given that the pixels corresponding to the background are far more numerous than the pixels of defects, a mask-guided stochastic generator for augmentation of industrial data could potentially yield improvements in detection and classification algorithms. To actually realize this gain, our model employs numerous strategies to produce controllable, realistic, and high-resolution synthetic industrial images as well as to enhance the quality of images and stabilize the training process. We propose a new GAN architecture that maps a given mask input to the sample space more efficiently by coupling the mask embedding vector, conditional label vector, and latent noise vector. Compared with the traditional image-to-image translation GANs described above, the samples generated by the GAN model are more diverse.

In the context of industrial images with surface defects, recently, several GAN-based methods have been proposed. One of these methods is Mask2Defect GAN [30], which proposes a GAN model to generate a large volume of surface defect images with different features and shapes. The algorithm separates the generation process into two steps: the first step uses the mask-to-defect construction network (M2DCNet) to render the defect details according to the binary mask, and the second step uses the fake-to-real domain transformation GAN (F2RDT-GAN) to add background textures and transform the synthesized defects from the rendered domain to the real defect domain.

Another method is the surface defect generative adversarial network (SDGAN) [31], which utilizes D2 adversarial loss and cycle-consistency loss to generate high-quality and diverse defect datasets using a small number of defect images. SDGAN incorporates two diversity control discriminators and a cycle-consistency loss to generate defect images in a more efficient and effective way. The introduction of the diversity control discriminators allows one to control the diversity of the generated images, while the cycle-consistency loss helps to ensure that the generated images are consistent with the input images. Defect-GAN [32] is proposed to mimic defacement and restoration processes to generate realistic and diverse defect samples. Defect-GAN employs adaptive noise insertion to capture the stochastic variations within defects. Kaiqiong et al. [33] proposed an entirely multiscale GAN with a transformer to capture the intrinsic patterns of qualified samples of IC metal package images at multiple scales. The proposed GAN model is designed to improve the quality of the generated images by capturing the patterns of the images at multiple scales. The multiscale architecture is achieved by using a set of convolutional layers with different dilation rates to extract features at multiple scales. One of the key contributions of the proposed GAN model is the use of a Swin Transformer decoder, which is designed to strengthen the modeling ability of the GAN. The Swin Transformer decoder is a modified version of the transformer decoder that is designed to handle images with high resolution. Shuanlong et al. [34] proposed a novel approach for generating synthetic defects in metal surfaces that is based on the concept of image inpainting. The proposed method regards defect generation as a form of image inpainting, where defects are generated in nondefect images in regions specified by defect masks.

Our proposed method, Magna-Defect-GAN, is better than the abovementioned methods in several ways. One key advantage is that our method maps a given mask input to the sample space more efficiently by coupling the mask embedding vector, guide vector, and latent noise vector. This allows for the generation of various images that are realistic and captured under different thicknesses, brightness, and types of fasteners. In contrast, previous methods require significant manual effort to create masks with all possible combinations of defect parameters, such as defects with different thicknesses, brightness, etc. Our method involves learning a disentangled representation to separate the different elements of fastener images, such as the parameters of defects and the background. This makes it useful for creating a large number of different defects, with varying thicknesses, brightness, and backgrounds, by simply adjusting the guide vectors. Another advantage of our method is that we propose to utilize the latent noise vector in addition to the guide vector to improve the diversity of generated images. This allows for the generation of images with different variations with respect to defects of different thicknesses, brightness, and types of fasteners.

## 3. Materials and Methods

We require a method for pixel-level data augmentation to increase the intraclass variety of the training dataset and strengthen the robustness of defect detection models with limited data. The mask-to-image translation model, which creates images that resemble those obtained in a different setting (lighting, texture, etc.), is a key part of our suggested methodology. We first suggest the guide vector in the GAN model, which contains values that are understandable by humans and is hence controllable and explicable. Using input from the guide vector and mask, we then synthesize images with significant intraclass variance. Finally, we use synthetic data to supplement the training dataset and develop a reliable defect detection model. In this section, we first present a novel dataset of fastener defects. Next, we present the Magna-Defect-GAN model and the guiding vector.

### 3.1. Line Scan Defect Dataset

**Figure 2:**
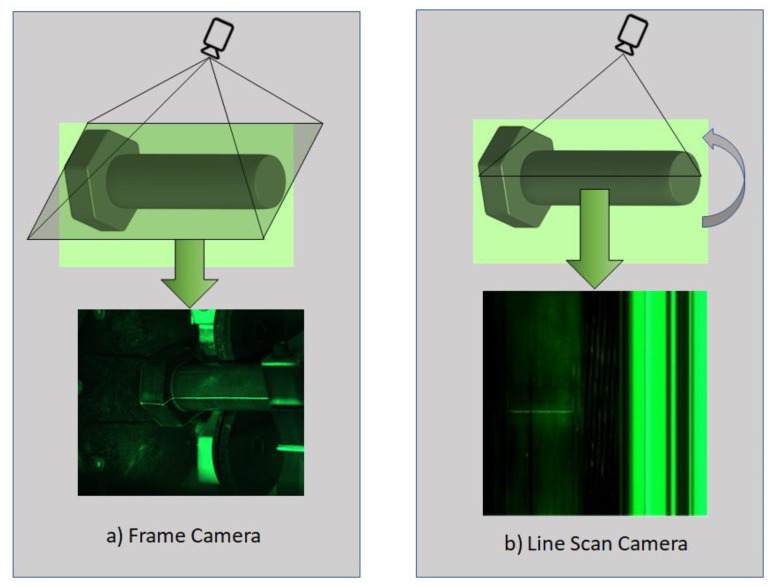
Comparison of (**a**) Frame and (**b**) Line Scan Cameras in terms of how they capture images.

We collected a new fastener defect dataset using a DALSA Linea 2k 7.04 um 2048 × 2−26 kHz-Color line scan camera since the frame cameras have their limitations in resolution and high-speed imaging applications. Unlike a frame camera, which exposes the entire area of the sensor and gives an entire image, a line scan camera exposes just a single line of pixels. These single lines of pixels are stitched together to form a complete frame. As opposed to frame cameras, line scan cameras need special optic systems. We used 12 mm fixed focal length lenses and 600 mm field of view (FOV). In our study, the linear array camera was used to capture MPI images because it allows for a larger field of view and a higher resolution compared to an area array camera. Additionally, linear array cameras have higher sensitivity and signal-to-noise ratio, which is important for detecting small defects. Furthermore, linear array cameras can also provide a higher frame rate, which is useful in fast-moving production lines. Additionally, linear array cameras are more cost effective and have a smaller form factor as compared to area array cameras. Figure 2 compares the methods used by frame and line scan cameras for image capturing.

**Figure 3:**
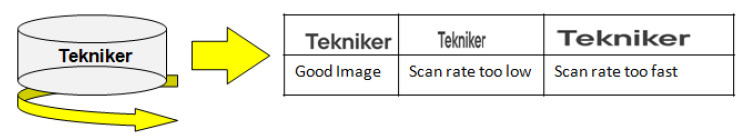
An illustration of scan rate not matching with moving speed.

The line scan camera must capture images at precisely the same rate that the fastener is being rotated—a too-fast scan rate, the image gets distorted; too slow, and some of the original slices are missed. We used an encoder to synchronize the rotational movement of the fastener as well as the triggering of the line scan camera to ensure that no unnecessary stretching or shrinking happens on the resultant image (Figure 3).

The collected defect dataset consists of 1050 RGB images. The dataset was collected from a magnetic particle inspection apparatus located at Erreka Fastening solutions. The images were collected in different environments (background, lighting, thickness, and brightness). Ground truth masks and guide labels were labeled by experienced quality engineers. Specifically, three components of defect images were annotated so that not only defect shape, location, and numbers but also the thickness, brightness, and background of the defects can be controlled. At the right end of the line scan image, we can see a sizable dark green region that represents the background, which was stationary.

### 3.2. Fine-Grained Guide Label

In general, the thickness and brightness of the defect in an image depend on (1) particle concentration, (2) lighting, and (3) material type of the fasteners (background). Since our goal is, given a mask label, generating various images that are realistic and captured under different thicknesses, brightness, and types of fasteners, we propose to utilize thickness, brightness, and background (guide vector) to guide an image generation process. Therefore, given a mask label and a guide vector ($e\;\in R^{3}$), a corresponding image is generated.

### 3.3. Preliminaries

GANs were introduced to make a generative model by having two models (generative model G and discriminative model D) compete with each other. A generative model G turns noise into an imitation of the data to try to trick the discriminator, and a discriminative model D tries to identify real data from fakes created by the generator. Both G and D could be a convolutional neural network. To create a synthetic image x, the generator takes a noise vector from a prior noise distribution $p_{z}\left(z\right)$ and runs it through a differentiable function: G(z)→x. The learning procedure of GAN is to train a discriminator D and a generator G in parallel. At each iteration, backpropagation is applied to adjust generator model parameters G to minimize log(1−D(G(z))) and adjust discriminator model parameters D to minimize logD(x). Therefore, the loss function of GANs can be written as:

(1) $$L_{\mathrm{GAN}}\left(G,D\right)={\;E}_{x\sim P_{r}\left(x\right)}\left[\mathrm{logD}\left(x\right)\right]+{\;E}_{z\sim P_{z}\left(z\right)}\left[\log\left(1-D\left(G\left(z\right)\right)\right)\right]$$

To have a control on the kind of image being generated, in cGANs, both the generator G and discriminator D are conditioned on additional information such as class labels y. In pix2pix GAN, both the generator G and discriminator D are conditioned on an input image to generate a corresponding output image. In this case, adversarial loss can be formulated as:

(2) $$L_{\mathrm{cGAN}}\left(G,D\right)={\;E}_{x\sim P_{r}\left(x\right)}\left[\mathrm{logD}\left(x|y\right)\right]+{\;E}_{z\sim P_{z}\left(z\right)}\left[\log\left(1-D\left(G\left(z|y\right)\right)\right)\right]$$

### 3.4. Proposed Architecture

#### 3.4.1. Generator Architecture

**Figure 4:**
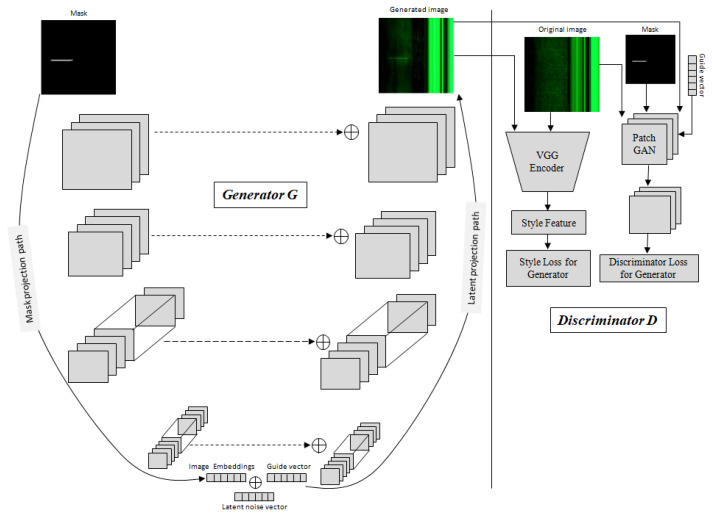
The Magna-Defect-GAN model comprises a U-net style generator network, a discriminator network, and a pretrained VGG feature extractor. The mask projection path in the Generator network G is tasked with mapping input masks into image embeddings through encoder blocks. The latent projection path is used to translate the combination of the image embeddings, guide vectors, and latent noise vectors into the output image. The discriminator network D is trained to distinguish real and generated images. The pretrained VGG network is used to extract features to calculate the style loss.

The main challenge to training an image-to-image translation GAN without latent noise vector z is that the model would produce deterministic outputs. Wang et al. [35] used latent noise vector z as an input to the generator model in addition to the mask label. To improve the overall feature projection efficiency, our Magna-Defect-GAN model first performs mask embedding in the generator before the latent projection layer. Figure 4 represents the overall architecture of our Magna-Defect-GAN. The generator of our proposed GAN is based on a U-Net style design that can be decomposed into two branches, namely, the mask projection and the latent projection branch. First, the mask projection branch encodes the input mask into the mask embedding (32-dimensional vector). This mask projection branch consists of 7 convolution layers each with a stride of 2, each followed by a leaky rectified linear unit (Leaky ReLU). After that, we concatenate latent noise vector z (132-dimensional vector) and the guide label vector e with the mask embedding to improve sample space mapping and provide diverse texture detail in the synthetic images. Finally, the latent projection branch whose inputs are the latent noise vector z, which, in combination with the mask embedding and guide label, generates an output image. An image mask input provides the intended defect shape, position, and quantity, and a guide label provides the necessary defect background and thickness to generate a defect image.

#### 3.4.2. Discriminator Architecture

We employed a modified Patch-GAN architecture [26] for the discriminator. As opposed to classifying the output and target image as being real or fake, the Patch-GAN discriminator is designed to use a convolutional network that divides the input images into NxN patches of the image and outputs a matrix of values. Consequently, the discriminator gives feedback on each region or patch of the image, which enables high frequency and encourages detailed outputs by the generator. To avoid the common tiling artifacts with smaller patch sizes, 70 × 70 patches are typically used. However, we found that smaller patches in combination with style transfer losses yield sharper images while eliminating tiling artifacts. Consequently, we use a patch size of 16 × 16.

#### 3.4.3. Loss Function

One of the key elements of GANs is the loss function that is used to train the GAN models. Different types of loss functions can be used depending on the specific GAN architecture and the desired properties of the generated samples.

The pix2pix loss function is commonly used in image-to-image translation tasks, such as converting a sketch to an image or converting a daytime image to a nighttime image. The main goal of the pix2pix loss function is to generate an output image that is as similar as possible to the target image. To achieve this, the pix2pix loss function uses two main components: the L1 loss and the adversarial loss.

The L1 loss, also known as the mean absolute error (MAE), compares the pixel-wise differences between the generated image and the target image. It calculates the absolute difference between each pixel in the generated image and the corresponding pixel in the target image and then takes the average of all these differences. The L1 loss is a popular choice for image-to-image translation tasks because it is less sensitive to outliers than the L2 loss (mean squared error) and has been shown to produce sharper images. The L1 loss is calculated as:

(3) $$L_{1}=E_{x,y,z}∥x-G{(z,y)∥}_{1}$$

where x is the target image and G(z,y) is the generated image. The L1 loss is a good choice for image-to-image translation tasks because it is able to capture the structural information of the image.

The pix2pix GAN loss is used in combination with the L1 loss to ensure that the generated image is not only similar to the target image but also visually realistic. The total loss function for the pix2pix method is a combination of the L1 loss and the adversarial loss. The total loss function is defined as:

(4) $$L_{pix2pix\;}=\;\alpha \;*\;L_{1}\;\mathrm{loss}\;+\left(1-\alpha \right)*L_{adv}$$

where α is a hyperparameter that controls the balance between the L1 loss and the adversarial loss.

The CycleGAN loss function, on the other hand, uses a combination of the cycle-consistency loss and the adversarial loss. The cycle-consistency loss, also known as the cycle-consistency constraint, ensures that the generated image can be transformed back to the original image. The cycle-consistency loss is calculated as the difference between the original image and the transformed image. The cycle-consistency loss is defined as:

(5) $$L_{Cycle}=E_{x}∥x-G{(F\left(x\right))∥}_{1}+E_{y}∥y-F{\left(G\left(y\right)\right)∥}_{1}$$

where x is the input image, y is the target image, G is the generator for the input image, and F is the generator for the target image. The cycle-consistency loss ensures that the generated image preserves the characteristics of the input image.

Adversarial loss is used to ensure that the generated image looks like a real image and not a fake one. The total loss function for the CycleGAN method is a combination of the cycle-consistency loss and the adversarial loss. The total loss function is defined as:

(6) $$L_{CycleGAN\;}=\lambda \;*\;L_{Cycle}+L_{adv}$$

where λ is a hyperparameter that controls the balance between the cycle-consistency loss and the adversarial loss.

We used a combination of three different losses in our proposed GAN model, i.e., adversarial loss, style loss, and reconstruction loss.

- Adversarial loss is used to ensure that the generated images are realistic and not easily distinguishable from the original images. This is done by training the generator to fool the discriminator, which is trained to distinguish between real and fake images.
- Style loss is used to ensure that the generated images have the same style as the original images. This is done by comparing the feature maps of the generated images to the feature maps of the original images.
- Reconstruction loss is used to ensure that the generated images are similar to the original images. This is done by comparing the generated images to the original images.

By combining these three types of losses, the GAN is able to generate high-quality images that have the same style and structure as the original images while also being realistic and difficult to distinguish from real images. This results in more realistic and visually appealing generated images.

The adversarial loss in the Magna-Defect-GAN is essential to ensure and guide the generator to generate synthetic images that look real and are able to fool the discriminator. The generator establishes a relationship between the source image mask y, guide vector e, and the random noise image z to the target image x, i.e., y,z,e →x. The discriminator makes a distinction between original and fake x| y,e. The adversarial loss can be represented as:

(7) $$L_{adv}=E_{y,e,\;x}\left[\mathrm{logD}\left(y|e,x\right)\right]+{\;E}_{z,y,e}\left[\log\left(1-D\left(y,e,G\left(z,y|e\right)\right)\right)\right]$$

The conditional adversarial loss attempts to make the generated image look real. However, line scan industrial images, different from natural images that have higher diversity in texture, shape, and color, require intricate precision of internal structure. As a result, an additional constraint is necessary to ensure that the generated images are similar to the original. Therefore, we add a pixel-wise reconstruction loss to the adversarial loss that measures the pixel-wise distance between the generated images and the original image that is available at training time. Comparing the performance of utilizing L1 and L2 norms to ascertain the reconstruction loss, we observe that the L2 norm appears to perform better for our task. Our reconstruction loss is defined as below:

(8) $$L_{rec}=E_{y,e,x}∥x-G{\left(z,y,e\right)∥}_{2}^{2}$$

The abovementioned reconstruction loss ensures structural consistency between the generated and original image. To make the training process more stable and minimize the total textural deviation between the generated and original image, style loss could be used as auxiliary regularization. We use the style loss to further improve the similarity between the generated and the original image in terms of intricate visual appearance such as texture and color. We employ style loss at multiple levels between the original and the generated image with a pretrained VGG model in a way similar to an earlier work [36]. The style information is measured as the degree of correlation between feature maps in a given layer. The style loss is then calculated by matching the mean and standard deviation between the feature maps computed by the generated image and the original image. We calculate the pair-wise correlation between all the feature vectors in the filters for each style layer in order to preserve similarity between the style image and the generated image based on the spatial information. These feature correlations are given by Gram matrix $G^{l}\in \;{\mathbb{R}}^{N_{l}\times N_{l}}$, the inner product between the vectorized feature maps in layer l:

(9) $$G_{ij}^{l}=\overset{M_{l}}{\underset{k=1}{\sum }}F_{ik}^{l}F_{jk}^{l}$$

Assume that there are $A_{l}$ filters in total, each with a feature map of size $B_{l}$, and that we have $G_{\mathrm{ij}}^{l}$, $H_{\mathrm{ij}}^{l}$ gram matrices for the style image and the generated image. Thus, we can calculate the overall style loss as follows:

(10) $$L_{\mathrm{style}}=\underset{l}{\sum }w_{l}.\frac{1}{4A_{l}^{2}B_{l}^{2}}\underset{i,j}{\sum }{\left(G_{i,j}^{l}-H_{i,j}^{l}\right)}^{2}$$

where the weight given to layer l is $w_{l}$. Each $w_{l}$, in this case, contains the value $\frac{1}{\mathrm{Total}\;\mathrm{number}\;\mathrm{of}\;\mathrm{style}\;\mathrm{layers}},\;i.e.,\;\frac{1}{5}$.

Total generator loss is calculated as a weighted combination of reconstruction loss, style loss, and adversarial loss. Our final generator loss is formulated as:

(11) $$L_{\mathrm{total}}={\;\lambda }_{1}L_{\mathrm{adv}}+\lambda_{2}L_{\mathrm{rec}}+\lambda_{3}L_{\mathrm{style}}$$

where $\lambda_{1}$, $\lambda_{2}$, and $\lambda_{3}$ regulate the relative weight of different loss terms. Although reconstruction should take precedence during the optimization phase, the adversarial loss plays a significant role in encouraging local realism of the synthesized output in our mask-to-image translation problem. We conducted our studies with the following settings: $\lambda_{1}=10$; $\lambda_{2}=\lambda_{3}=0.1$.

## 4. Experiments

### 4.1. Training Details

All the experiments were run on Google-cloud infrastructure using a single Nvidia 12 GB Titan X GPU. We randomly divided our data into 80% training set and 20% test set. We trained the GAN model for an average of 200 epochs, and the generation of synthetic images took about 0.0265 ms per image. We kept the learning rate constant for the first 100 epochs, after which it exponentially declined to zero during the following epochs. All weights in the model were initialized from a Gaussian distribution with a mean of 0 and standard deviation of 0.01.

### 4.2. Evaluation Metrics

When evaluating the GAN model, it is important to consider not only fidelity, which quantifies the quality of the generated images, but also the diversity. In other words, fidelity measures image quality and diversity measures the variety of the generated images. A good generator must produce a good variety of images. For instance, of all images in the training dataset, the generator should model all types of defects, including defects in different positions, shapes, and sizes. We use the Inception score (IS) and Fréchet Inception Distance score (FID) to evaluate the performance of our Magna-Defect-GAN model. IS attempts to measure both the fidelity and diversity of the generated images. The Inception model pretrained on a fine-grained defect dataset is the backbone of the IS. Given image x and label y, for a high fidelity and diverse input, the posterior probability of a label p(y|x) computed using the Inception model should have a low entropy, and the marginal class distribution ∫P(y|x=G(z)) dz should have a high entropy. Mathematically, IS can be represented as:

(12) $$\mathrm{IS}\left(G\right)=\exp\left(E_{x\sim p_{a}}D_{\mathrm{KL}}\left(p\left(y|x\right)∥p\left(y\right)\right)\right)$$

FID is the frequently used method to measure the feature distance between real and generated images. Images from the training dataset and images generated by the generator are transformed into a feature space by FID using the output of the last hidden layer in Inception Net. Multivariate normal Fréchet Distance can be calculated as:

(13) $$\mathrm{FID}\left(x,g\right)=∥\mu_{x}-\mu_{g}∥{}_{2}^{2}+\mathrm{Tr}(\Sigma_{x}+{\;\Sigma }_{g}-2{\left(\Sigma_{x}\Sigma_{g}\right)}^{\frac{1}{2}}$$

where $\left(\mu_{x},\mu_{g}\right)$ and $\left(\Sigma_{x},\Sigma_{g}\right)$ represent the mean and covariance of the true and generated features, respectively.

### 4.3. t-SNE Visualization

To provide more powerful evidence that the generated synthetic images, indeed, contribute to the shape of the data manifold, we use a t-distributed stochastic neighbor embedding (t-SNE) algorithm to visualize the distribution of training and generated image samples by reducing high-dimensional data to a 2D plane. First, the t-SNE algorithm converts the similarities between data points to joint probabilities and then aims to minimize the KL divergence between the joint probability of the low-dimensional embedding and the high-dimensional data.

### 4.4. Evaluation of Defect Classification

In order to assess the performance benefits obtained by utilizing GAN-based synthetic images, we benchmark our approach with well-known existing CNN models, ResNet [37] and EfficientNet [38], which are frequently in a number of defect classification applications. The defect classification performances are compared for the following training approaches. (a) Model trained only with the original dataset, (b) model trained with the augmented dataset (traditional augmentation methods such as rotation, vertical/horizontal flips, zoom, shear, and channel shifts), (c) model pretrained with synthetic dataset and fine-tuned with the original dataset, (d) model pretrained with the ImageNet dataset and fine-tuned with the augmented dataset, (e) model pretrained with the ImageNet dataset and fine-tuned with the synthetic dataset, and (f) model pretrained with the ImageNet dataset and fine-tuned with a mix of augmented and synthesized datasets. The metrics employed for the performance comparison are precision, recall, F1 score, and binary accuracy.

(14) $$\mathrm{Precision}=\frac{\mathrm{TP}}{\mathrm{TP}+\mathrm{FP}}$$

(15) $$\mathrm{Recall}=\frac{\mathrm{TP}}{\mathrm{TP}+\mathrm{FN}}$$

(16) $$\mathrm{F1}\;\mathrm{Score}=\frac{2.(\mathrm{precision}.\mathrm{Recall})}{\left(\mathrm{precision}+\mathrm{Recall}\right)}$$

(17) $$\mathrm{Accuracy}=\frac{\mathrm{TP}+\mathrm{TN}}{\mathrm{TP}+\mathrm{FP}+\mathrm{TN}+\mathrm{FN}}$$

where TP, TN, FP, and FN denote true positive (correctly identified defects), true negative (correctly identified nondefect images), false positive (images erroneously classified as defect), and false negative (images erroneously classified as nondefect), respectively.

## 5. Results and Discussion

**Figure 5:**
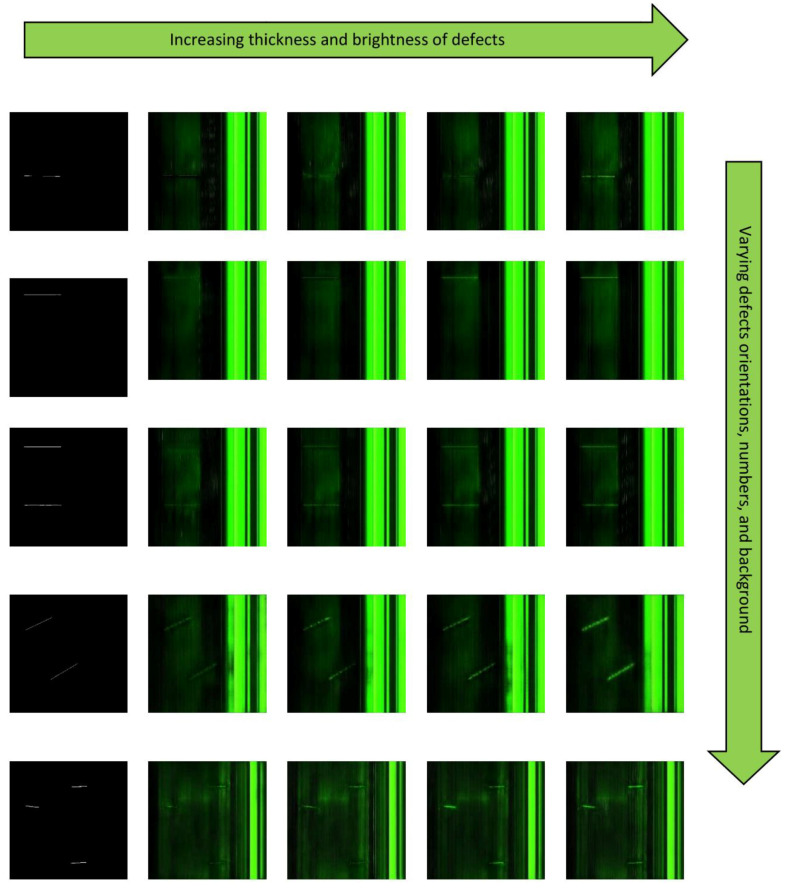
Manipulating Guide vectors on defect dataset. The different rows correspond to different Guide vector settings of fixed mask input and noise.

The Magna-Defect-GAN model was trained using 780 nondefective images and 270 defective images. After training the model, diverse and realistic synthetic images were generated by altering the input masks and guide vector. Because of the small training sample size, the augmented images by rotation, vertical/horizontal flips, zoom, and shear were additionally incorporated. We present the synthesized images given a mask and guiding vectors in Figure 5 to illustrate the controllability and explainability of the Magna-Defect-GAN model.

**Figure 6:**
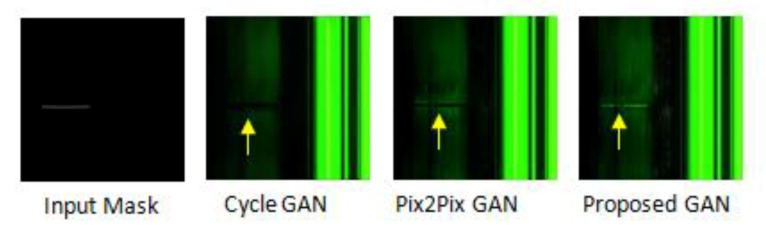
Comparison between the Magna-Defect-GAN and different image translation approaches.

Figure 5 demonstrates how the proposed method can generate different fine-grained images from a given mask by altering the guide vectors. The first column in Figure 5 depicts the input masks, while the second column depicts the GAN-generated images given guide vectors. As illustrated in Figure 6, the generated images of the Magna-Defect-GAN are almost identical to the training dataset with high-fidelity image-specific details well preserved (e.g., defects, illumination, and background), while Pix2Pix and CycleGAN show poor perceptual quality.

It is worth noting that other GAN models would potentially generate a number of completely blank pixels on a defect region (Figure 6) in an effort to achieve greater diversity. This is harmful for training a defect detection model since the model would struggle to learn from the noisy images. However, our Magna-Defect-GAN model maintains both structural consistency and fine-grained background details, which is beneficial for a defect detection model to learn from different appearances of defects under different levels of ambient lighting.

**Figure 7:**
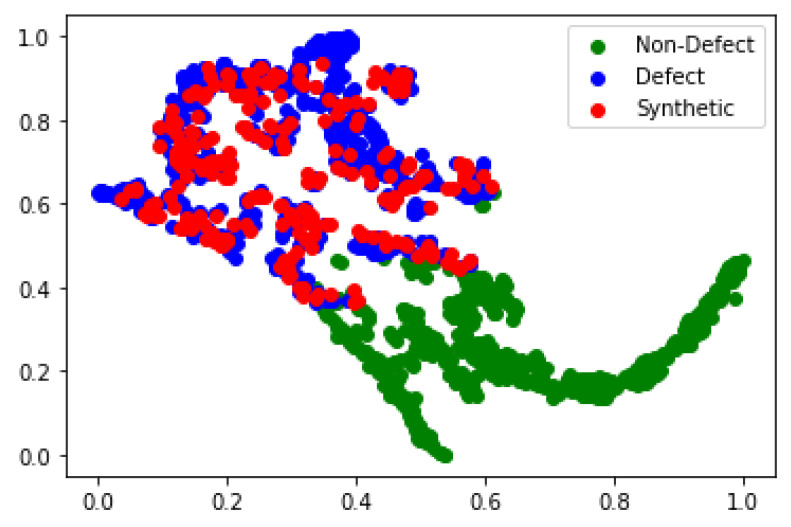
t-SNE visualization showing the effect of the Magna-Defect-GAN-based augmentation.

Figure 7 shows the data distribution of original and synthetic images after dimensionality reduction by the t-SNE algorithm. As this figure illustrates, the proposed algorithm can generate data that not only overlap with the true data distribution but is also extremely close to the underlying distribution of the training data. All the generated data that mimic the real data distribution have not appeared in the training dataset. The proposed algorithm can provide an efficient way to close gaps in the discrete manifold distribution and supplement sources of variance that are difficult to augment in conventional methods.

Our proposed method achieves better IS and FID scores (Table 1) compared to the rest of the methods. There could be a couple of reasons for the better scores. First, coupling the mask embedding vector, conditional label vector, and latent noise vector results in better sample space mapping, which leads to diverse textures of fine-grained details in the synthesized images. Moreover, the use of style loss in the Magna-Defect-GAN improves the fidelity of the generated image in terms of background attributes such as texture and color.

We investigated the use of GAN-generated images to supplement the training dataset for the task of defect classification in the presence of a small number of training examples. Our Magna-Defect-GAN model was employed for generating photorealistic and high-resolution synthetic industrial images.

Using our proposed method, it is possible to generate images with large intraclass variations such as defects with different thicknesses, brightness, types of fasteners, etc. Furthermore, it is feasible to create realistic synthetic images that are similar to the training data by using simulated masks with different forms, locations, and orientations. Table 2 summarizes the six sets of experiments that were carried out to compare different training schemes. The defect classification accuracy and F1 score by the Resnet model trained with the original data from scratch were 80.8% and 0.801, respectively. The efficientnet-B7 model accuracy and F1 score were 89.8% and 0.893, respectively. When the training dataset was augmented by applying random but realistic lighter data augmentation schemes such as vertical/horizontal flips, zoom, and rotations, the model accuracy and F1 score of the Resnet model were enhanced to 83.5% and 0.868, respectively. Additionally, by incorporating the efficientnet-B7 model, the accuracy and F1 score further improved to 91.8% and 0.918, respectively. Because the training data size is too small and does not contain enough data samples to properly represent the greatest possible intraclass diversity, using the original data samples alone resulted in low and unstable training and validation accuracy. The training loss stabilized when the lighter data augmentation scheme was applied; however, the validation loss remained unstable. The accuracy and F1 score of the Resnet model were 87.5% and 0.88, respectively, using synthetic images for pretraining and the original dataset for fine-tuning. Additionally, the efficientnet-B7 model achieved an accuracy of 92.5% and an F1 score of 0.925 when using the same pretraining and fine-tuning methods. Compared with the results of the training model with the augmented dataset, the test accuracy and F1 score were comparable. The accuracy and AUC of the Resnet model increased from 83.5% to 89.8% and 0.868 to 0.919, respectively, when the ImageNet pretrained model was used and fine-tuned with the augmented dataset. Additionally, the efficientnet-B7 model showed an improvement in accuracy, increasing from 91.8% to 94.7%, and an increase in F1 score from 0.918 to 0.946. This is because the ImageNet dataset spans 21,000 object classes, the model is encouraged to learn more features than it requires when it is pretrained with fine-grain labels, and these excess features aid in network generalization, i.e., improving the testing accuracy. In other words, fine-grain labels help learn more features than coarse-grained labels (defect vs. nondefect).

When the synthetic images were used for fine-tuning, the accuracy and the F1 score of the Resnet model were 92.7% and 0.939, respectively, using the ImageNet pretrained model. The efficientnet-B7 model achieved an accuracy of 96.4% and an F1 score of 0.966 when fine-tuned with the synthetic images. The performance of defect detection models using synthetic images is on par with the outcome of regular data augmentation. It is observed that when the model was fine-tuned with a mix of synthetic and augmented data, the accuracy and the F1 score of Resnet were 93.5% and 0.947, respectively, which is clearly a better performance rate. Similarly, when using the efficientnet-B7 model, the accuracy and F1 score were even higher at 97.2% and 0.973, respectively, further demonstrating the effectiveness of using a mix of synthetic and augmented data in fine-tuning models. Both traditional augmentations and GAN-generated synthetic images are extremely beneficial to prevent overfitting when training a defect detection model with a limited dataset—the former extrapolates the training data distribution and the latter generates more diverse data by interpolating between the discrete data points in the manifold.

## 6. Conclusions

In this work, we addressed the problem of defect detection with limited data. For that purpose, we proposed a GAN-based mask-to-image translation model for data augmentation. The main conclusions are listed below.

(1) By combining the mask embedding vector with the latent noise vector and the discrete fine-grained guide labels, an improved conditional mask-to-image translation GAN was proposed. Synthetic images with large intraclass diversity such as defect size, shape, position, thickness, brightness, and background can be generated conditionally.
(2) The proposed model training process was more stable, and the generated data sample was of higher quality when compared to the existing GAN models.
(3) GAN-based augmentation is a useful tool for bridging holes in the discrete training data distribution and enhancing sources of intraclass variation that are challenging to amplify in other ways, but they cannot expand the distribution beyond the training dataset extremes.
(4) When training a defect detection model with a small dataset, a mix of conventional augmentations and GAN-generated synthetic images are extremely helpful to avoid overfitting. The conventional data augmentation extrapolates the training data distribution, while the GAN-based synthetic images add more diversity by interpolating between the discrete data points in the manifold.

## Figures and Tables

**Table 1:** Performance of the Magna-Defect-GAN Model.

Model	Inception Score ↑	FID ↓
Cycle [29]	2.88 ± 0.25	91.56
Pix2Pix [26]	3.08 ± 0.31	65.09
**Magna-Defect-GAN**	**3.88 ± 0.36**	**50.03**

**Table 2:** Data Augmentation Experiments on Surface Defect Dataset.

Training Scheme	Model	Recall	Precision	F1-Score	Accuracy
a	ResNet [37]	0.684	0.969	0.801	0.808
	EfficientNet-B7 [38]	0.847	0.945	0.893	0.898
b	ResNet [37]	0.873	0.865	0.868	0.835
	EfficientNet-B7 [38]	0.895	0.943	0.918	0.918
c	ResNet [37]	0.863	0.907	0.88	0.875
	EfficientNet-B7 [38]	0.909	0.942	0.925	0.925
d	ResNet [37]	0.942	0.898	0.919	0.898
	EfficientNet-B7 [38]	0.958	0.935	0.946	0.947
e	ResNet [37]	0.945	0.934	0.939	0.927
	EfficientNet-B7 [38]	0.961	0.972	0.966	0.964
**f**	**ResNet** [37]	**0.955**	**0.94**	**0.947**	**0.935**
	**EfficientNet-B7** [38]	**0.969**	**0.977**	**0.973**	**0.972**

## Data Availability

The data presented in this study are available upon request from the corresponding author.
